# Long-term impact of delayed follow-up due to COVID-19 lockdown on patients with neovascular age-related macular degeneration

**DOI:** 10.1186/s12886-022-02453-4

**Published:** 2022-05-20

**Authors:** Stephan Szegedi, Christian Ebner, Kata Miháltz, Tobias Wachter, Pia Veronika Vécsei-Marlovits

**Affiliations:** 1 Department of Ophthalmology, Clinic Hietzing, Vienna Healthcare Group, Vienna, Austria; 2 Karl Landsteiner Institute for Process Optimisation and Quality Management in Cataract Surgery, Vienna, Austria

**Keywords:** Neovascular age-related macular degeneration (nAMD), Coronavirus disease 2019 (COVID-19), Anti-vascular endothelial growth factor (anti-VEGF)

## Abstract

**Background:**

During the first wave of the coronavirus disease 2019 (COVID-19) pandemic in 2020 outpatient care of neovascular age-related macular degeneration (nAMD) patients was severely reduced due to lockdown. Missed visits are known to be detrimental to patients in need of continued anti-vascular endothelial growth factor (VEGF) intravitreal injections (IVIs). The purpose of the study was to assess the effect of a month-long pause of regular visits and anti-VEGF IVIs in nAMD patients.

**Methods:**

A retrospective study was performed. Patients were treated in a pro re nata (“as needed”) scheme. Distance (logMAR) and near (logRAD) visual acuity (VA), optical coherence tomography, delay between planned and actual visit date and the indication for IVI were assessed for 3 continous visits in the 6 months before lockdown (V-3, -2, -1) and the 2 visits after lockdown (V0, V + 1). For analysis of long-term impact, records for visits 1 years before and after lockdown (V-3, V + 2) were gathered.

**Results:**

We included 166 patients (120 female, 46 male) with a median (range) age of 80.88 (59.8–99.36) years. Compared to V-1, distance VA was significantly worse at both V0 (0.27 ± 0.21 vs 0.31 ± 0.23 logMAR, *p* < 0.001) and V + 1 (0.27 ± 0.21 vs 0.30 ± 0.23 logMAR, *p* = 0.021). Near VA was significantly worse at both V0 (0.31 ± 0.21 vs 0.34 ± 0.22 logRAD, *p* = 0.037) and V + 1 (0.31 ± 0.21 vs 0.34 ± 0.22 logRAD, *p* = 0.02). Visit delay (VD) at V0 was significantly longer than at V + 1 (30.81 ± 20.44 vs 2.02 ± 6.79 days, *p* < 0.0001). Linear regression analysis showed a significant association between visit delay and a reduction of near VA between V-1 and V + 1 (*p* = 0.0223). There was a significant loss of distance VA (*p* = 0.02) in the year after the lockdown period (*n* = 125) compared to the year before. Loss of reading acuity was not significantly increased (*p* = 0.3). One year post lockdown, there was no correlation between VA change and visit delay after lockdown (*p* > 0.05).

**Conclusions:**

In nAMD patients whose visits and treatment were paused for a month during the first wave of the COVID-19 pandemic, we found a loss of VA immediately after lockdown, which persisted during follow-up despite re-established anti-VEGF treatment. In the short term, length of delay was predictive for loss of reading VA. The comparison of development of VA during the year before and after the lockdown showed a progression of nAMD related VA loss which may have been accelerated by the disruption of regular visits and treatment.

**Trial registration:**

This article does not report the outcome of a health care intervention. This retrospective study was therefore not registered in a clinical trials database.

## Background

The first wave of the coronavirus disease 2019 (COVID-19) pandemic led to lockdowns in many countries in the first half of 2020 in an effort to limit the spreading of the disease and the resulting burden on health care systems. Accordingly, the volume of outpatient visits was reduced by delaying planned visits and asking patients to only visit outpatient units in urgent cases. Patients suffering from neovascular age-related macular degeneration (nAMD) were particularly affected by these developments: as an elderly patient group, they are at higher risk for severe COVID-19 illness and should be protected from possible nosocomial transmission of the disease, on the other hand, the standard of care for active nAMD are regular anti-vascular endothelial growth factor (VEGF) intravitreal injections (IVIs) [1]. In the European Union, the first vaccine against COVID-19 was authorised for use in December 2020. From March 16, 2020 to April 14, 2020, all non-emergency procedures, including anti-VEGF IVIs, were paused at our department. After this period, treatment was resumed according to medical need [2]. It has been previously reported that missed or delayed visits are associated with worse outcomes for patients with nAMD [3, 4]. To determine the long term impact of delayed follow-up visits and treatment due to first wave COVID-19 lockdown on patients with neovascular age-related macular degeneration, a retrospective study was conducted.

## Methods

We included patients with a diagnosis of choroidal neovascularisation (CNV) in at least 1 eye due to neovascular age-related macular degeneration (nAMD) who had 3 or more visits (visit (V) -3, -2, -1) in the 6 months before March 17, 2020, history of at least 1 anti-VEGF IVI between March 17, 2020 and May 17, 2020, 2 or more visits between March 17, 2020 and August 17, 2020 (V0, V + 1). For analysis of long-term impact, patients with a visit approximately 1 year (at least 10 months, as close as available to 12 months) before (V-4) and after the lockdown (V + 2) were included.

All patients were treated in a pro re nata (PRN, “as needed”) scheme.

If patients had a diagnosis of CNV due to nAMD for both eyes, the eye with worse VA was chosen as study eye; where VA was equal in both eyes, the eye that had received the higher number of previous IVIs was chosen; where the number of previous IVIs was equal for both eyes, the right eye was chosen.

Patients with best spectacle corrected visual acuity (VA) worse than 1.00 logMAR (logarithm of the minimum angle of resolution) in both eyes or eye disease relevant to VA other than choroidal neovascularisation were excluded.

Decimal distance VA was converted to letters logMAR, near VA was measured in logRAD (logarithmic reading acuity determination) using Radner reading charts (Oculus Optikgeräte, Wetzlar, Germany) [5] and converted to letters logRAD. CRT was measured within a 1 mm radius circular area using optical coherence tomography (OCT, Heidelberg Spectralis OCT, Heidelberg Engineering, Heidelberg, Germany).

Change between visits was calculated as difference for VA and percent change for CRT. Time to visit (TV) was calculated as days passed since the previous visit. For CNV patients receiving bevacizumab, the due visit date at our institution is 4 weeks after IVI, for patients receiving aflibercept, 6 weeks after IVI. Visit delay (VD) was calculated as days difference between due and actual visit date.

Statistical analysis was performed in R (version 3.6.3, R Foundation for Statistical Computing, Vienna, Austria) [6]. The level of significance was set at *p* < 0.05.

## Results

### Short-term impact

We included 166 patients, 120 (72.3%) female, 46 (27.7%) male, with a median (range) age of 80.88 (59.80–99.36) years. Baseline data including counts of anti-VEGF drugs received are summarised in Table 1.

**Table 1:** Baseline data at V0, values are given as means (standard deviation) or percent where indicated

			Missing
n		166	
Sex (%)	f	120 (72.3)	0.0
	m	46 (27.7)	
Age (years, median [range])		80.88 [59.80, 99.36]	0.0
Disease duration (years, median [range])		2.94 [0.16, 13.67]	0.6
Total IVI (median [range])		15.00 [1.00, 62.00]	0.6
Avastin IVI (median [range])		12.00 [1.00, 42.00]	2.4
Eylea IVI (median [range])		4.00 [1.00, 18.00]	38.0
Lucentis IVI (median [range])		4.00 [1.00, 21.00]	61.4

**Fig. 1:**
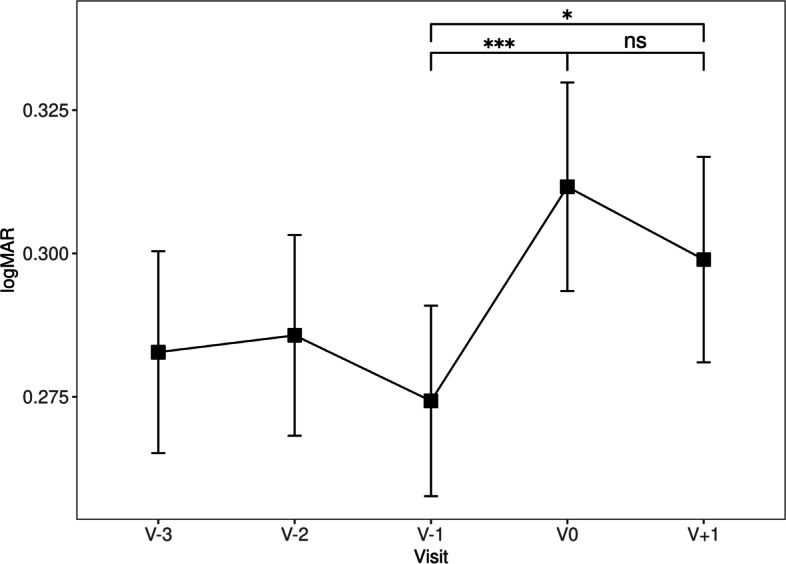
VA (logMAR) over time, error bars represent standard error of the mean, ns not significant (*p* ≥ 0.05), * *p* < 0.05, *** *p* < 0.001. (*n* = 166)

**Fig. 2:**
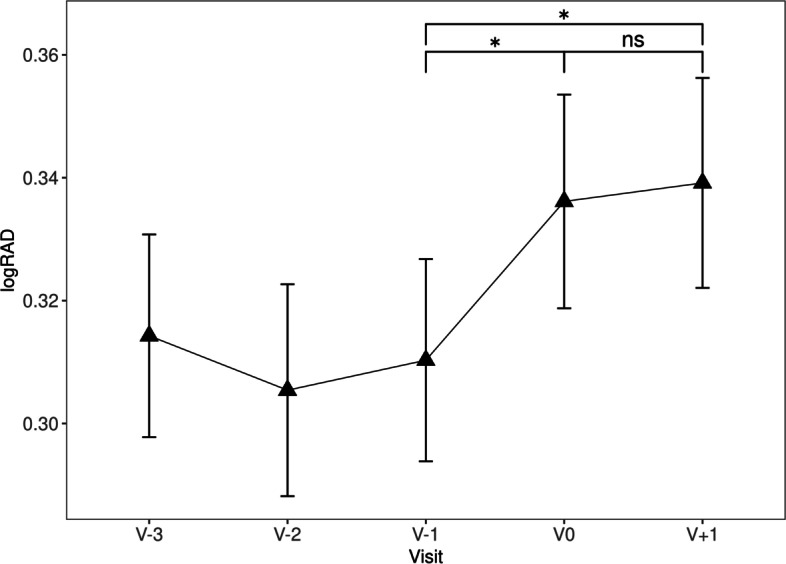
Near VA (logRAD) over time, error bars represent standard error of the mean, ns not significant (*p* ≥ 0.05), * *p* < 0.05. (*n* = 166)

Compared to V-1, distance VA was significantly worse at both V0 (0.27 ± 0.21 vs 0.31 ± 0.23 logMAR, *p* = 0.000648) and V + 1 (0.27 ± 0.21 vs 0.30 ± 0.23 logMAR, *p* = 0.021). No significant difference in distance VA was found between V0 and V + 1 (0.31 ± 0.23 vs 0.30 ± 0.23 logMAR, *p* = 0.142, Table 2, Fig. 1). Similarly, compared to V-1, near VA was significantly worse at both V0 (0.31 ± 0.21 vs 0.34 ± 0.22 logRAD, *p* = 0.037) and V + 1 (0.31 ± 0.21 vs 0.34 ± 0.22 logRAD, *p* = 0.02) than at V-1, but not significantly different between V0 and V + 1 (0.34 ± 0.22 vs 0.34 ± 0.22 logRAD, *p* = 0.744, Table 2, Fig. 2).

**Table 2:** Variables over time, values are given as means (standard deviation) or percent where indicated

	V-3	V-2	V-1	V0	V + 1
VA (logMAR)	0.28 (0.23)	0.29 (0.23)	0.27 (0.21)	0.31 (0.23)	0.30 (0.23)
VA (logMAR): change to previous visit	—	0.00 (0.11)	-0.01 (0.09)	0.04 (0.11)	-0.01 (0.09)
Near VA (logRAD)	0.31 (0.21)	0.31 (0.22)	0.31 (0.21)	0.34 (0.22)	0.34 (0.22)
Near VA (logRAD): change to previous visit	—	-0.01 (0.11)	0.00 (0.09)	0.03 (0.11)	0.00 (0.09)
CRT (µm)	340.89 (101.84)	342.70 (102.83)	335.12 (98.46)	361.60 (112.83)	336.87 (106.83)
CRT (µm): change to previous visit	—	2.87 (22.64)	-0.44 (18.44)	9.14 (21.86)	-5.63 (14.79)
SRF (%)	64 (38.6)	66 (39.8)	59 (35.5)	71 (42.8)	64 (38.6)
IRF (%)	75 (45.2)	76 (45.8)	88 (53.0)	87 (52.4)	74 (45.1)
IVI indicated at visit (%)	106 (63.9)	113 (68.5)	125 (75.3)	136 (82.4)	117 (70.5)
TV (days)	—	47.54 (13.97)	43.07 (11.25)	75.14 (19.29)	36.41 (7.63)
VD (days)	—	—	—	30.81 (20.44)	2.02 (6.79)

**Fig. 3:**
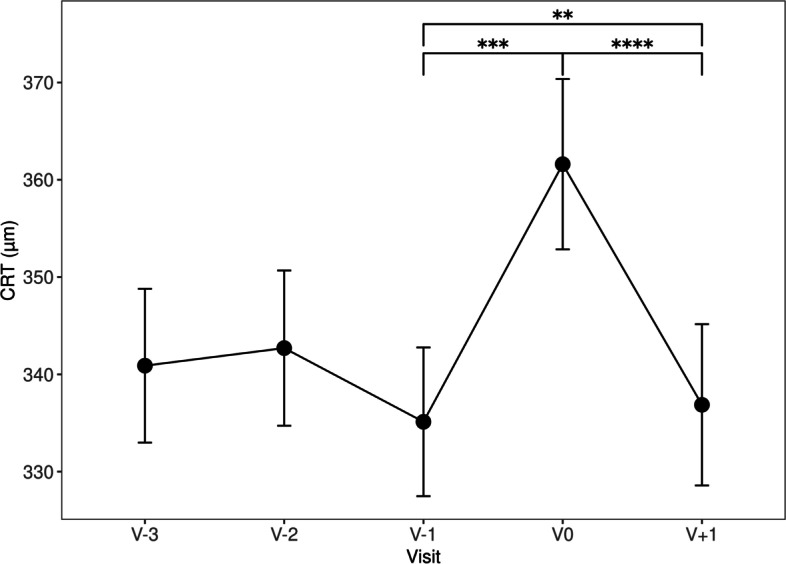
CRT (µm) over time, error bars represent standard error of the mean, ** *p* < 0.01, *** *p* < 0.001, **** *p* < 0.0001. (*n* = 166)

When compared to V-1, CRT was significantly greater at V0 (335.12 ± 98.46 vs 361.60 ± 112.83 µm, *p* = 0.00000678), but not significantly different at V + 1 (335.12 ± 98.46 vs 336.87 ± 106.83 µm, *p* = 0.683). At V + 1, CRT was significantly less than at V0 (336.87 ± 106.83 vs 361.60 ± 112.83 µm, *p* = 0.0000164, Table 2, Fig. 3).

Frequency of SRF and IRF was not significantly different between V-1 (35.5% SRF, 53.0% IRF) and V0 (42.8% SRF, *p* = 0.2; 52.4% IRF, *p* = 0.857), V0 and V + 1 (38.6% IRF, *p* = 0.496; 45.1% SRF, *p* = 0.0924), or V-1 and V + 1 (SRF: *p* = 0.496, IRF: *p* = 0.0924). Frequency of IVIs was not significantly different between V-1 and V0 (75.3% vs 82.4%, *p* = 0.262), but significantly higher at V0 than at V + 1 (82.4% vs 70.5%, *p* = 0.0199). Between V-1 and V + 1, there was no significant difference in the frequency of IVIs (*p* = 0.267, Table 2).

TV was 75.12 ± 19.29 days for V0, significantly longer than for V-1 (43.07 ± 11.25 days, *p* < 0.0001) or V + 1 (36.41 ± 7.63 days, *p* < 0.0001). TV was significantly shorter at V + 1 than at V-1 (*p* < 0.0001). VD at V0 was significantly longer than at V + 1 (30.81 ± 20.44 vs 2.02 ± 6.79 days, *p* < 0.0001, Table 2).

To assess the short-term influence of VD on VA loss, linear regression was performed. Linear regression analysis did not show a significant association between VD at V0 and reduction of distance VA between V-1 and V + 1 (*p* = 0.0754). However, length of VD was significantly associated with a reduction of near VA between V-1 and V + 1 (*p* = 0.0226, Table 3).

**Table 3:** Results of linear regression with visit delay (VD) at V0 as the predictor variable, * *p* < 0.05

Response variable	Regression coefficient	*p*
VA (logMAR): change from V-1 to V + 1	0.0008198	0.0754
Near VA (logRAD): change from V-1 to V + 1	0.0009814	0.0226*

### Long-term impact

**Fig. 4:**
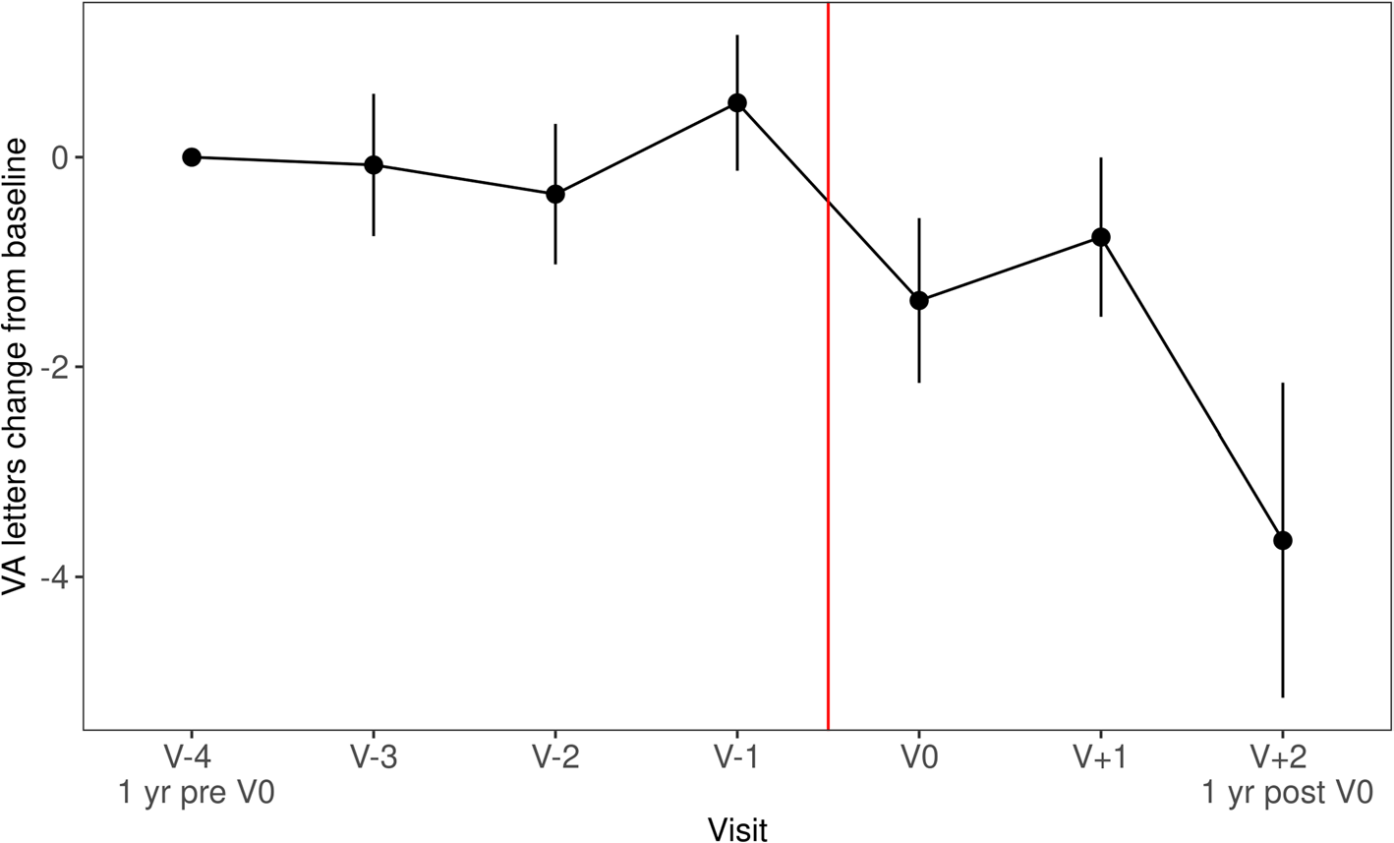
VA letters change from V-4 over time, red line indicates lockdown (*n* = 125)

**Fig. 5:**
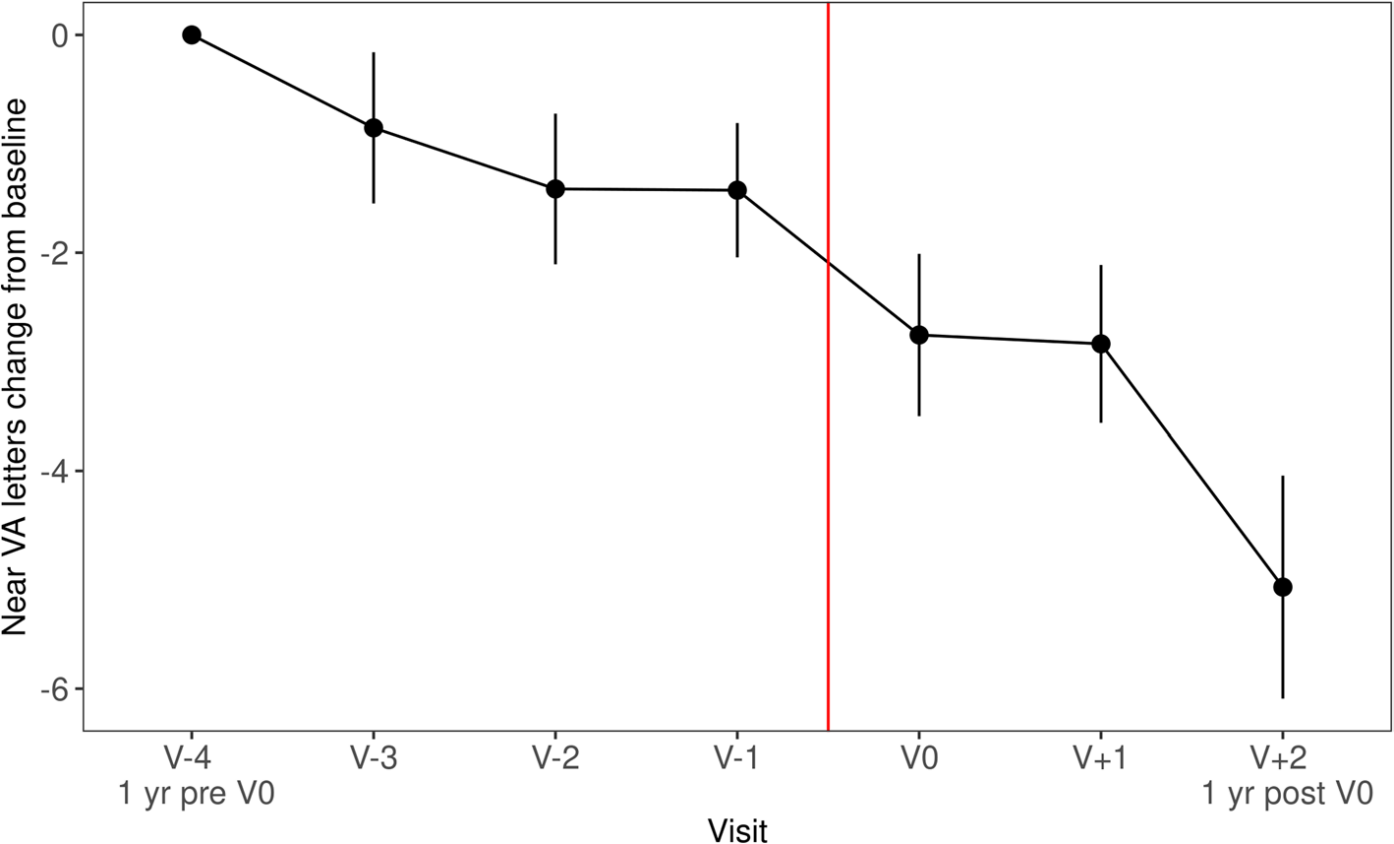
Near VA letters change from V-4 over time, red line indicates lockdown (*n* = 125)

To assess long-term impact of treatment disruption by the lockdown period, patients with visits 1 year before V0 and 1 year after V0 were selected for analysis (*n* = 125). The loss of VA between V-4 to V0 and V + 1 to V + 2 was compared using Wilcoxon signed rank tests with continuity correction. There was a significant increase in lost letters logMAR (*p* = 0.02) in the years after the lockdown period. However, the loss of letters logRAD was not significantly increased (*p* = 0.3, Figs. 4, 5).

One year post lockdown, there was no correlation between VA change and visit delay after lockdown, disease duration or age (Pearson correlation, *p* > 0.05). There was no difference in post-lockdown VA deterioration between patients with visits delayed for a short (< 14 days, *n* = 28), medium (14–30 days, *n* = 40) and long (> 30 days, *n* = 53) amount of time after lockdown (Kruskal–Wallis rank sum test, *p* = 0.33).

## Discussion

We studied the impact of delayed visits caused by a lockdown during the first wave of the COVID-19 pandemic on patients with CNV caused by nAMD. Of the patients included in the study, over 70% were female, a proportion close to pre-COVID-19-pandemic demographic data for patients receiving IVI for “degeneration of macula and posterior pole” (International Statistical Classification of Diseases and Related Health Problems—10th revision (ICD-10) diagnosis H35.3) at our clinic (65% female in 2019, internal data). After a mean visit delay of approximately 1 month, patients presented with significantly reduced distance and near VA, and significantly greater CRT. PRN treatment with anti-VEGF IVIs was resumed and at the next visit, patients presented on time and with significantly reduced CRT. VA, however, did not significantly improve compared to the visit after lockdown and was still significantly worse than at the last visit before lockdown. Linear regression showed the delay caused by the lockdown period to be a significant predictor of the reduction of near VA at the second visit after lockdown, even after IVI treatment had been continued.

These results are in accordance with reports predating the COVID-19 pandemic by Ramakrishnan et al. [3] and Greenlee et al. [4] who found that nAMD patients who missed visits and treatment had worse VA than patients presenting on time.

Several studies of the effect of the lockdown periods caused by the COVID-19 pandemic in the first half of 2020 on the treatment of nAMD patients have been conducted.

In a study of 100 patients with nAMD, Borrelli et al. [7] found that after a mean prolonged visit interval of 3.6 months, caused by the pandemic, patients had worse VA and a higher percentage of patients showed OCT evidence of disease activity. The time between visits was found to be a predictor of VA decrease after lockdown. Compared to our findings, Borrelli et al. observed a more prolonged visit interval, but similar decrease in VA. As the study did non include visits after the delayed visit, possible lasting effects of the delay could not be evaluated.

Yeter et al. [8] studied 106 patients with nAMD and found a decrease in VA, increased central macular thickness and a higher frequency of OCT evidence of disease activity after a lockdown period. Compared to our results, Yeter et al. found longer mean delay of visits and greater VA reduction. In contrast to our study, they did not find the length of delay between post-lockdown and last follow-up to be associated with VA reduction between pre-lockdown and last visit. At subsequent visits, OCT findings returned to pre-lockdown frequencies, but VA at the final visit, a mean 3.5 months later, did not improve compared to the post-lockdown visit.

In a study of 117 patients receiving regular IVIs, including 93 patients with nAMD, Naravane et al. [9] found reduced VA for patients with delayed visits, but not for patients presenting on time. However, the study only included two visits: the visit before declaration of the spring 2020 lockdown and the visit thereafter.

Stone et al. [10] conducted a review of patients receiving IVIs, including 537 patients with nAMD (166 delayed > 8 weeks, 518 not delayed or delayed < 8 weeks), and found that patients whose visits were delayed for more than 8 weeks suffered from a greater reduction in VA (mean change in VA: -5.18 letters) than patients whose visits were within 8 weeks of the due visit date. They found that at subsequent visits in 2020, 74.6% of the included nAMD patients had VA within 5 letters of baseline.

Examining the effects of a month-long visit pause (4 months minimum follow-up time post-lockdown) on nAMD patients in a treat and extend setting, Allegrini et al. found that in a cohort with a pre-lockdown visit interval of a mean 103 days, the observed VA loss of a mean 0.1 logMAR could not be clearly attributed to the 30-day visit interval increase [11].

In summary, our data are in good agreement with these previous results on short-term effects of missed visits and/or treatment for nAMD.

Stattin et al. [12] performed a retrospective study of 98 nAMD patients in a hospital in Vienna, Austria. While the initial loss of VA was comparable to our findings, contrary to our results, Stattin et al. found that visit delay remained a significant predictor of VA loss even 1 year after the lockdown.

The limitations of our study include the exclusion of patients with binocular vision worse than 1.00 logMAR, patients lost to follow-up after lockdown and lack of a control group. As OCT biomarker, we analysed CRT, IRF and SRF. The inclusion of other OCT biomarkers such as pigment epithelial detachment, alterations of outer retinal layers or hyperreflective foci could potentially help to identify patients at risk for accelerated disease progression.

The present study adds to the existing short-term evidence as it reports the long-term outcome of a unique month-long treatment interruption in a well-characterised cohort of pre-treated nAMD patients treated in a PRN scheme.

## Conclusions

In patients with CNV due to nAMD whose visits and treatment were paused for a month during the first wave of the COVID-19 pandemic, we found a loss of VA immediately after lockdown, which persisted during follow-up despite re-established anti-VEGF treatment. The length of the delay was predictive for loss of reading VA 1 month after reinstated visits. After 1 year however, length of visit delay was not predictive for the further decline of visual acuity.

Our results suggest that in patients with CNV due to nAMD, the prolonged visit interval due to the first-wave COVID-19 pandemic lockdown in early 2020 may have caused a progression of the disease with accelerated loss of VA compared to the pre-lockdown period—despite resumed treatment.

## Data Availability

Data for this study is not publicly available. Requests may be directed to the corresponding author.

## Abbreviations

COVID-19: Coronavirus disease 2019
nAMD: Neovascular age-related macular degeneration
VEGF: Vascular endothelial growth factor
IVI: Intravitreal injection
CNV: Choroidal neovascularisation
V: Visit
PRN: Pro re nata
VA: Visual acuity
logRAD: Logarithmic reading acuity determination
logMAR: Logarithm of the minimum angle of resolution
OCT: Optical coherence tomography
TV: Time to visit
VD: Visit delay
